# Three-Dimensional Visualisation of Burn Wounds: Concordance of Artec Eva and Revopoint Miraco with Clinical Photography—A Case Series

**DOI:** 10.3390/ebj7010007

**Published:** 2026-01-28

**Authors:** Katarína Dudová, Bibiána Ondrejová, Tomáš Demčák, Monika Michalíková, Lucia Bednarčíková, Jozef Živčák, Peter Lengyel, Erik Eliáš

**Affiliations:** 1 Department of Biomedical Engineering and Measurement, Faculty of Mechanical Engineering, Technical University of Košice, Letná 1/9, 042 00 Košice, Slovakia; bibiana.ondrejova@tuke.sk (B.O.); monika.michalikova@tuke.sk (M.M.); lucia.bednarcikova@tuke.sk (L.B.); jozef.zivcak@tuke.sk (J.Ž.); 2 Burns and Reconstructive Plastic Surgery Clinic, AGEL Hospital Košice-Šaca, Lúčna 512, 040 15 Šaca, Slovakia; tomas.demcak@nke.agel.sk (T.D.); peter.lengyel@nke.agel.sk (P.L.); erik.elias@nke.agel.sk (E.E.)

**Keywords:** three-dimensional imaging, burns, wound assessment, medical documentation, Artec Eva, Revopoint Miraco

## Abstract

Accurate documentation of burn wounds is essential for evaluating treatment outcomes and monitoring healing progression. Traditional two-dimensional (2D) photography remains the clinical standard but lacks depth and volumetric accuracy. Three-dimensional (3D) scanning offers enhanced visualization of wound morphology and tissue vitality, potentially improving objectivity in burn assessment. This study compares two handheld 3D scanning systems—Artec Eva and Revopoint Miraco—in documenting acute and healing burn wounds, using standard clinical photography as the reference. Fifteen patients with second-degree and third-degree burns were prospectively examined at the Burn Unit of AGEL Hospital Košice-Šaca, with five representative cases selected for detailed analysis. For each patient, clinical photographs and paired 3D scans were obtained under standardized conditions and evaluated for color fidelity, wound margin clarity, representation of epithelialisation islands, necrotic tissue, and correlation with clinical findings. Across all cases, Artec Eva demonstrated superior color accuracy, clearer wound delineation, and more realistic visualization of tissue vitality and re-epithelialisation. Revopoint Miraco reliably captured wound shape but produced darker tones and exaggerated surface relief, occasionally distorting depth perception. Overall, both systems successfully identified key healing features; however, Artec Eva provided more clinically accurate and visually consistent results. Three-dimensional scanning represents a valuable adjunct to conventional burn documentation.

## 1. Introduction

Accurate documentation and assessment of burn wounds are critical for guiding treatment, monitoring healing, and evaluating outcomes. Conventional two-dimensional (2D) photography remains standard in clinical settings, yet it fails to capture wound depth, surface irregularities, and volumetric changes essential for comprehensive analysis [1,2].

Three-dimensional (3D) imaging technologies have recently emerged as valuable tools for objective wound visualization and measurement. They enable precise evaluation of wound morphology, tissue vitality, and healing dynamics, offering higher reproducibility and more detailed topographical information than 2D methods [3,4,5]. Moreover, the integration of 3D imaging into burn rehabilitation—such as custom splints, masks, and facial orthoses—has expanded its clinical applications across reconstructive and scar management workflows [6,7].

Among the available systems, structured-light scanners such as Artec Eva provide high-resolution color accuracy and surface detail, while compact infrared-based devices like Revopoint Miraco offer faster, mobile data acquisition. Despite their increasing accessibility, comparative clinical studies assessing their performance in acute burn documentation remain limited [8,9,10].

This study aims to qualitatively compare two handheld 3D scanning systems—Artec Eva and Revopoint Miraco—in the visualization of acute and healing burn wounds. By correlating 3D scans with clinical photographs, we evaluate each system’s accuracy, color fidelity, and surface detail representation to determine their clinical utility in burn care.

## 2. Materials and Methods

To obtain patient data, ethical approval was first secured, and informed consent was obtained from each patient. The study was approved by the Ethics Committee of AGEL Hospital Košice-Šaca (Košice-Šaca, Slovakia) under the number 17-2023. A total of 15 patients with burn injuries or burn-related sequelae were prospectively monitored at burn unit in Agel Hospital Košice-Šaca.

For the purposes of this study, the analysis was intentionally limited to acute burns and actively healing burn wounds, as these clinical entities demonstrate clearly defined and temporally evolving morphological features that are particularly suitable for evaluating the visual performance and practical applicability of three-dimensional imaging systems in burn care. From the full dataset, five representative patients were selected for detailed qualitative analysis and presentation. The selection strategy was designed to capture a wide spectrum of burn characteristics, including variability in wound size, degree of burn injury (ranging from first- to third-degree burns), anatomical location, and different stages of the healing continuum. Visual assessment was performed by three physicians, who are also co-authors of this study. This approach enabled a comprehensive visual assessment of how each scanning system performs under diverse and clinically relevant conditions.

For each selected patient, conventional clinical photographs were obtained and directly paired with three-dimensional scans acquired within the same examination session. The study was designed as a purely visual and descriptive comparison of two handheld three-dimensional scanners based on different technological principles and representing substantially different cost and complexity categories. This design allowed the evaluation of whether a low-cost, portable scanning solution can provide visually comparable clinical information to that obtained from a high-end professional system.

The following two devices were used:

- Artec Eva (Artec 3D, Luxembourg): A structured-light scanner developed for high-resolution medical and industrial applications, offering sub-millimetre accuracy (up to 0.1 mm) and a scanning speed of 16 frames per second [8]. This device represents an established, high-cost (approximately EUR 15,000) professional standard in three-dimensional surface imaging.
- Revopoint Miraco (Revopoint 3D Technologies Inc., Shenzhen, China): A compact, infrared-based handheld scanner optimized for rapid mobile data acquisition, with an accuracy of up to 0.05 mm, scanning speeds of up to 15 frames per second, and a markedly lower acquisition cost (approximately EUR 1500), making it potentially accessible for broader clinical use [9].

The two three-dimensional scanning systems included in this study differ substantially in their underlying imaging principles, hardware design, and intended fields of application, which directly influence their performance and practical usability in a clinical burn care setting. The Artec Eva operates on structured-light technology, projecting a predefined light pattern onto the scanned surface and reconstructing geometry based on its deformation. This approach provides highly stable surface reconstruction and consistent spatial accuracy, particularly for larger anatomical regions, but requires controlled environmental conditions, external power supply, and dedicated post-processing software-Artec Studio (Artec 3D, Luxembourg). In contrast, the Revopoint Miraco utilizes infrared-based scanning with integrated onboard processing, enabling fully handheld and cable-free operation, with data acquisition and processing performed using Revo Scan software (Revopoint 3D Technologies Inc., Shenzhen, China). While this technology prioritizes portability and rapid acquisition, it may be more sensitive to surface reflectivity, variable tissue coloration, and subtle patient movement. These fundamental differences result in distinct trade-offs between geometric robustness, colour fidelity, acquisition flexibility, and cost, which are particularly relevant when considering the applicability of three-dimensional imaging in routine clinical workflows and resource-limited settings.

All scans were performed under controlled lighting conditions (approximately 500 lx of standard clinical ambient lighting) during routine wound dressing changes. Patients were positioned to ensure optimal visibility of the burn wounds while minimizing body movement and maintaining patient comfort. Each wound area was scanned twice, once with each scanner, with only minimal patient repositioning between sessions. The scanning distance ranged from 0.5 m to 1 m. On average, the complete acquisition process, including scanning and generation of the 3D model, required approximately 4–6 min when using the Revopoint Miraco, whereas the corresponding workflow with Artec Eva was generally faster. In parallel, photographic documentation was acquired at the same time points and within the same distance range as the scanning procedure to ensure consistency between imaging modalities.

The resulting 3D models were evaluated qualitatively with emphasis on wound-margin clarity, representation of colour and tissue vitality, visibility of epidermal loss, necrotic surfaces, epithelialisation islands, crust formation, and overall visual correspondence with clinical photography. No quantitative parameters, such as wound surface area or volumetric measurements, were included in the analysis, as the primary objective of this study was to assess the visual descriptive capabilities of two three-dimensional scanning systems with markedly different technological sophistication and economic requirements.

## 3. Results

A total of 15 patients with burns or burn scars of various etiologies, stages, and extents have been monitored, and the case studies of five selected patients are presented in the current article.

### 3.1. Subject 1

The first case was of a 42-year-old man who was transported to the emergency department on 9 April 2025 with a burn injury located on the dorsal aspect of the left foot. The patient was subsequently admitted to the burn unit, where he received comprehensive local and systemic treatment.

**Figure 1:**
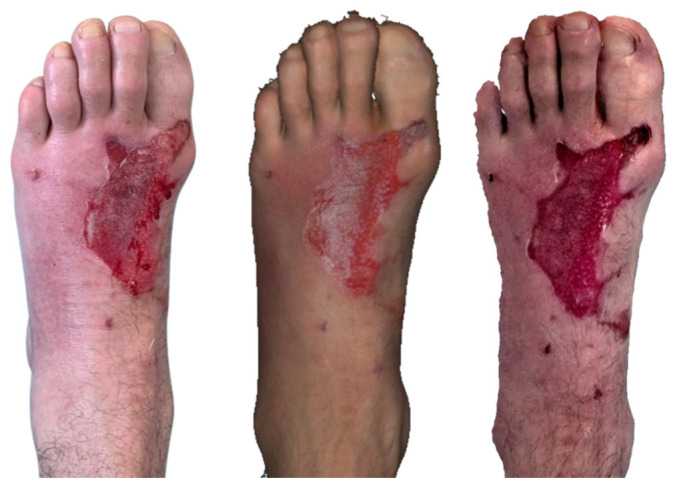
Photograph (**left**), Artec Eva 3D scan (**middle**), and Revopoint Miraco 3D scan (**right**) of the burn on the following day after its occurrence.

Clinical photography and 3D scanning of the affected area were performed on the following day on 10 April 2025 (Figure 1). On the dorsal aspect of the patient’s left foot, a partial thickness burn injury consistent with a second-degree superficial partial-thickness grade is evident. The central zone exhibits a visibly deeper thermal injury, characterized by a pale, moist surface typical of injured but not fully necrotic dermal structures. Along with the lateral margin, a delicate yet clearly defined rim of necrotic tissue can be distinguished, suggesting localized progression towards deeper tissue compromise.

The 3D reconstruction acquired with Artec Eva reveals an even broader distribution of avital dermis than is readily apparent in clinical photography. Particularly on the medial side, areas of preserved, vital dermis are rendered with high chromatic fidelity. In general, Artec Eva provides more accurate colour reproduction and finer differentiation of tissue vitality when compared with Revopoint Miraco.

In contrast, the Revopoint Miraco scan displays a predominantly viable dermal surface over much of the affected area. Only subtle avital zones are noted laterally, while the medial region demonstrates surfaces already undergoing re-epithelialisation. These distinctions highlight differences in colour sensitivity and depth perception capabilities between the two scanning systems.

**Figure 2:**
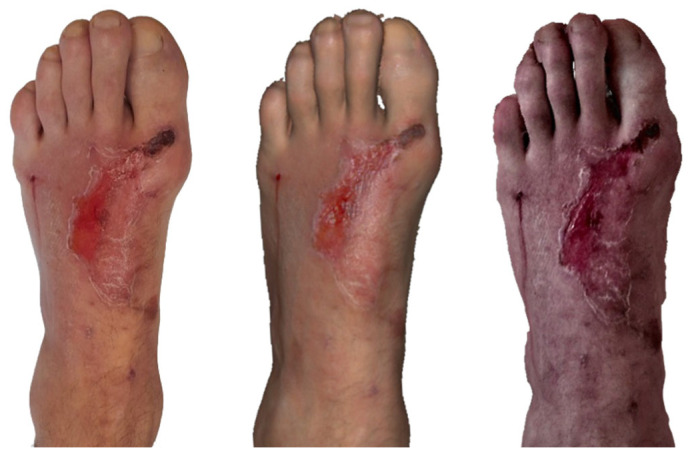
Photograph (**left**), Artec Eva 3D scan (**middle**), and Revopoint Miraco 3D scan (**right**) of the burn on the 5th day after its occurrence.

By the follow-up examination on 14 April 2025, day 5 post-injury (Figure 2), on the dorsal side of the patient’s left foot, the burn wound shows clear signs of spontaneous healing. Laterally, a residual defect measuring approximately 4 cm^2^ persists, characterized by a healthy granulating base. Medially, the tissue has already undergone complete re-epithelialisation, forming a smooth, delicate surface indicative of successful regenerative processes. Distally, a dry crust remains adherent, corresponding to the final stage of superficial wound maturation.

The Artec Eva scan depicts a slightly larger lateral residual area of approximately 5 cm^2^, yet the morphology and distribution of granulation tissue correspond closely to the clinical examination. The distal crust is represented with similar dimensions, and the medial re-epithelialised zone aligns well with the photographic documentation.

The Revopoint Miraco scan also identifies a residual wound measuring roughly 5 cm^2^. Within this region, subtle central hypergranulation is suggested, manifesting as a mild elevation in surface texture on the 3D model. The overall extent of the healed medial area is comparable to that in both the clinical image and the Artec Eva reconstruction, supporting consistency in wound size evaluation across modalities.

### 3.2. Subject 2

A 52-year-old male patient was acutely admitted to the Burn Care unit with burn injuries. On Wednesday, 23 April 2025, while burning branches, an aerosol can exploded in the fire, causing splashing and flame exposure. The patient received initial treatment at the Dermatology Department, followed by further management at the local Surgical Department. He subsequently presented to our burn outpatient clinic on his own. Given the extent and depth of the burns, at −8% TBSA, and with a first- and second-degree superficial partial thickness, the patient was admitted on 27 April 2025 for acute care in Burns and Reconstructive Plastic Surgery Clinic AGEL Hospital Košice Šaca. He underwent regular dressing changes, with all affected areas deemed suitable for conservative management, and he participated in intensive rehabilitation. During hospitalization, ENT and ophthalmology consultations were performed, with findings as noted above. At the time of discharge, all burn areas were fully healed, re-epithelialised, and without residual defects.

**Figure 3:**
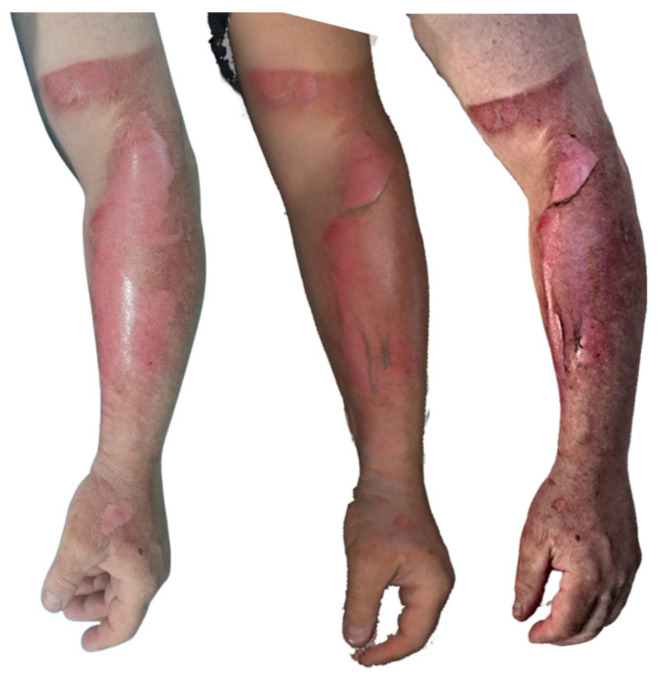
Photograph (**left**), Artec Eva 3D scan (**middle**), and Revopoint Miraco 3D scan (**right**) of the burn on the 5th day after its occurrence.

On 29 April 2025, A clinical photograph and scans (Figure 3) were taken, in which a second degree superficial partial thickness burn of the left forearm is visible, with detached epidermis covering approximately 2% of total body surface area.

The Artec Eva 3D scan depicts the same burn injury; however, epidermal detachment appears less extensive compared with that in the clinical photograph, with only about 1% TBSA lacking epidermis.

The Revopoint Miraco scan shows a distribution of preserved epidermis similar to that seen with the Artec Eva device. The remaining areas appear as healed or already re-epithelialised surfaces, suggesting advanced progress of spontaneous healing.

**Figure 4:**
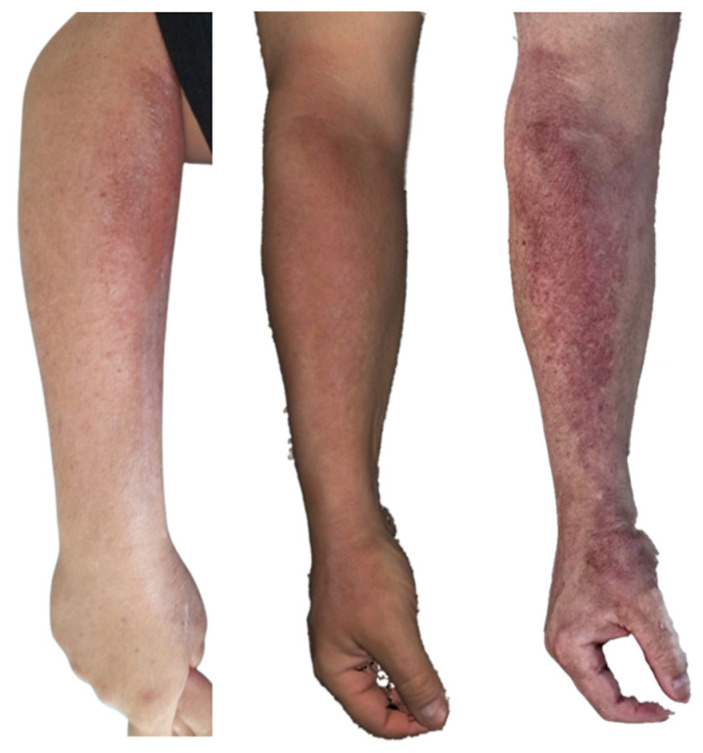
Photograph (**left**), Artec Eva 3D scan (**middle**) and Revopoint Miraco 3D scan (**right**) of the burn on the 5th day after its occurrence.

On the clinical photograph (Figure 4), a healed burn injury is visible on the right forearm, with central erythema present.

The Artec Eva scan shows the same healed burn area; however, the skin appears mildly erythematous over a slightly larger surface compared with that in the clinical image.

On the Revopoint Miraco scan, the right forearm displays a red–violet texture, which resembles the appearance typically seen after a dermo-epidermal skin graft. This finding does not correlate with the actual clinical condition, suggesting a colour-rendering or texture-interpretation artefact of the scanner.

### 3.3. Subject 3

On 21 May 2025, 77-year-old man sustained burn injuries while burning leaves and grass. When he poured gasoline onto the fire, the fuel ignited along the stream, producing a flash flame that caused second-degree, deep partial-thickness burns involving approximately 8% of his total body surface area, affecting the right upper limb, part of the back, and the thigh.

**Figure 5:**
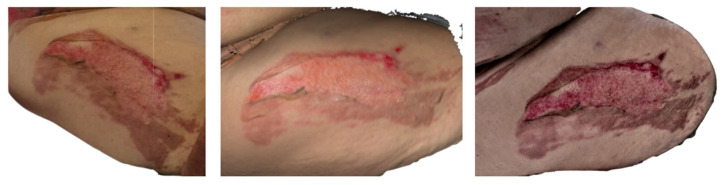
Photograph (**left**), Artec Eva 3D scan (**middle**) and Revopoint Miraco 3D scan (**right**) of the burn on the 2nd day after its occurrence.

The clinical photograph of the area on day 2 after the injury (Figure 5) shows a clearly demarcated burn wound on the ventrolateral aspect of the right thigh. Approximately 1% of the total body surface area demonstrates complete loss of the epidermis, exposing a pale, evenly contoured wound bed. Along the lateral margin, the epidermis remains preserved, creating a distinct and sharp transition between viable and injured tissue.

The Artec Eva scan captures the same region, with epidermal loss involving roughly 1% TBSA, revealing a comparably pale wound base. The extent and distribution of preserved epidermis correspond closely with the clinical photograph, suggesting faithful surface representation and accurate anatomical mapping.

The Revopoint Miraco scan likewise depicts a burn area with epidermal absence over approximately 1% TBSA. The wound bed appears pale in several regions, interspersed with patches of vital, red-hued tissue, yielding a more heterogeneous, subtly variegated texture. This pattern creates a slightly different visual impression compared with both the clinical photograph and the Artec Eva reconstruction.

**Figure 6:**
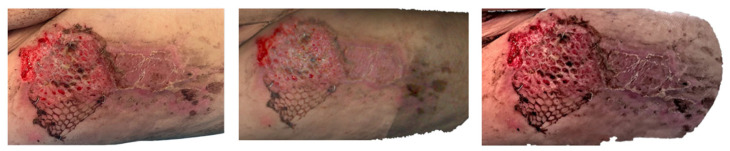
Photograph (**left**), Artec Eva 3D scan (**middle**), and Revopoint Miraco 3D scan (**right**) of the burn on the 23rd day after its occurrence.

In the clinical photograph taken on 13 June 2025 (Figure 6), the proximal portion of the wound shows the condition after a meshed dermo-epidermal graft (DE graft). The graft is centrally avital, secured with staples, while the segment extending beyond the wound margins onto intact skin appears dry and desiccated. The wound bed beneath is vital and red, indicating good perfusion. The distal part of the burn is already re-epithelialized, forming a smooth, healed surface.

The Artec Eva scan demonstrates the meshed DE graft with excellent visual clarity. The central section is rendered as avital, the overhanging portion appears dry, and the distal region is re-epithelialised, closely matching the clinical photograph.

The Revopoint Miraco scan similarly provides a clear depiction of the meshed DE graft. The central area appears avital, and the segment extending beyond the wound bed is shown as noticeably dry. The distal area is re-epithelialised, with a greater amount of dry surface skin captured in the reconstruction—an appearance that correlates well with that in the clinical image.

**Figure 7:**
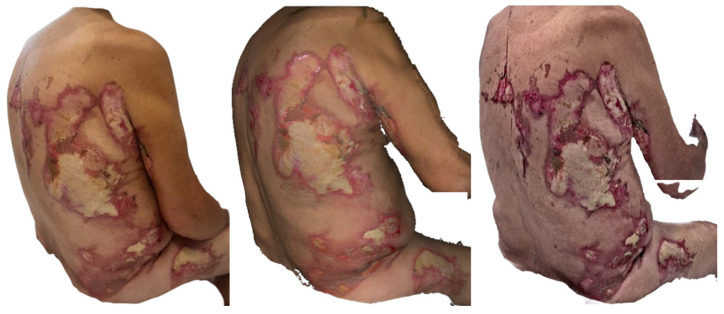
Photograph (**left**), Artec Eva 3D scan (**middle**) and Revopoint Miraco 3D scan (**right**) of the burn on the eighth day after its occurrence.

On 29 May 2025, the clinical photograph taken eighth day post-injury shows second-degree deep partial-thickness to third-degree full-thickness burns involving the back and the lateral aspect of the right thigh. Across nearly the entire burned surface, white necrotic coverings are visible, indicating non-viable tissue. Along the periphery, red, vital areas representing second-degree superficial partial-thickness burns can be observed. In the axillary region of the back, there is a small area with a thin necrotic layer, through which the red, viable wound bed is partially visible (Figure 7).

The Artec Eva scan depicts burn injuries consistent with the second and third degrees, with extensive white necrotic surfaces seen throughout most of the affected areas. The region on the back near the axilla, where a second-degree superficial partial-thickness burn is present, correlates well with the photographic findings.

The Revopoint Miraco scan likewise shows grade second-degree deep partial-thickness to third-degree full-thickness burns, with visible white necrotic coverings. However, in some regions, these necrotic layers appear slightly thinner, allowing subtle translucency of the underlying vital tissue to be appreciated, creating a somewhat different visual impression compared with that in both the photograph and the Artec Eva scan.

### 3.4. Subject 4

A 20-year-old male patient was admitted to our clinic via an air rescue service after sustaining extensive burn injuries on 31 May 2025. The burns involved the face, upper extremities, torso, and lower extremities, affecting 43% of the total body surface area (TBSA), with second-degree superficial partial-thickness and deep partial-thickness burns, following an indoor explosion that occurred while cleaning a computer with compressed air.

Upon arrival, the patient was intubated and transferred to the Burn Intensive Care Unit. Thermal injury to the airways was subsequently excluded. The patient was later extubated, and throughout the remainder of his hospitalization remained hemodynamically and clinically stable. All burn surfaces were managed conservatively, left to undergo spontaneous healing, and the patient completed a course of hyperbaric oxygen therapy (HBO) as well as intensive rehabilitation. At the time of discharge, only minimal residual defects were still present, primarily on the right shoulder, which were left to heal spontaneously. The patient was discharged home in stable condition and was thoroughly instructed regarding ongoing outpatient care and follow-up.

In the clinical photograph taken on 10 June 2025, 10 days post-injury, second-degree superficial partial-thickness burns are visible on the right upper limb, most prominently on the shoulder. The affected area shows epidermal loss with the presence of fine necrotic coatings. The forearm is fully re-epithelialised, displaying a smooth, healed surface.

The Artec Eva scan demonstrates second-degree superficial partial thickness burns on the right upper limb. On the shoulder, a well-demarcated area showing signs of re-epithelialisation can be observed, while the region proximal to the pink, vital surface contains necrotic tissue remnants. The forearm appears re-epithelialised, in accordance with the clinical image.

**Figure 8:**
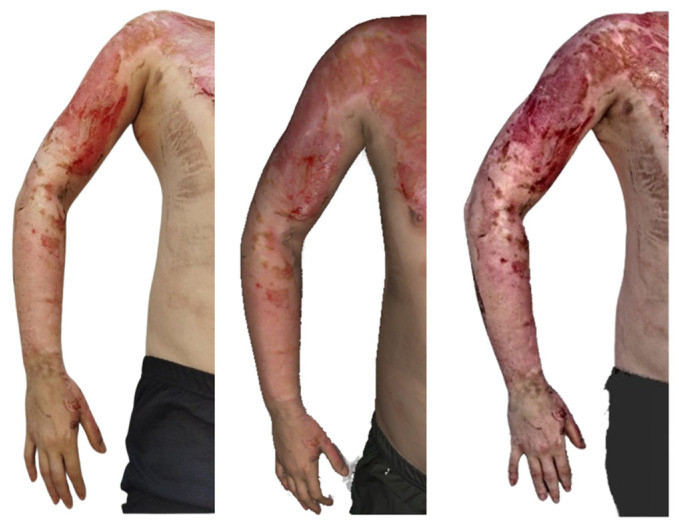
Photograph (**left**), Artec Eva 3D scan (**middle**), and Revopoint Miraco 3D scan (**right**) of the burn on the 10th day after its occurrence.

The Revopoint Miraco scan likewise depicts second-degree superficial partial-thickness burns. On the shoulder, areas with absent epidermis are evident, accompanied proximally by necrotic surface deposits. The forearm is re-epithelialised, consistent with both the clinical photograph and the Artec Eva reconstruction (Figure 8).

**Figure 9:**
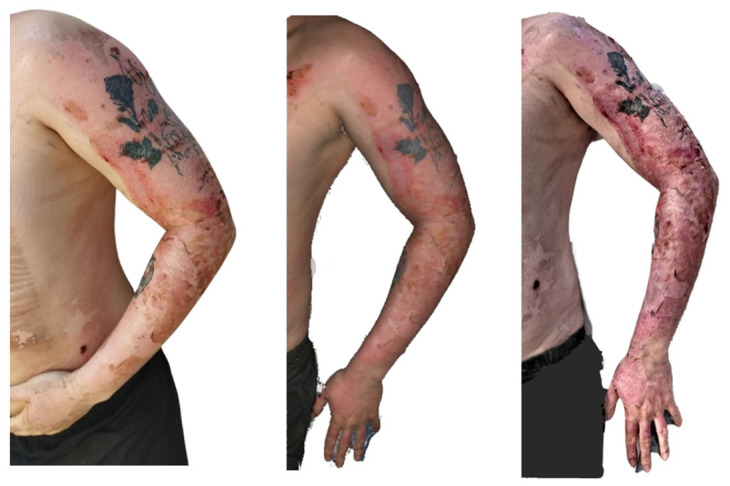
Photograph (**left**), Artec Eva 3D scan (**middle**), and Revopoint Miraco 3D scan (**right**) of the burn on the 10th day after its occurrence.

On the left upper limb (Figure 9), second-degree superficial partial thickness burns are present, extending from the shoulder down to the wrist. Tattoos are visible on both the shoulder and the forearm. The burned areas appear largely re-epithelialised, with a distal shoulder region showing a red, residual wound left to heal spontaneously.

The Artec Eva scan shows burn surfaces in the stage of re-epithelialisation, with the residual defect on the shoulder corresponding well to that in the clinical photograph. The texture of the tattoo is accurately reproduced and correlates with that in the photographic image.

In contrast, the Revopoint Miraco scan depicts second-degree superficial partial-thickness and deep partial-thickness burns on the left upper limb. Certain areas appear as though the surface relief were hypertrophic, creating an impression inconsistent with that in the photograph. The defect on the shoulder shows a necrotic coating in its distal portion, and the overall appearance does not correlate with the clinical findings.

### 3.5. Subject 5

A 21-year-old male patient was transported by helicopter from Poprad and acutely admitted to the clinic with extensive burn injuries involving the face, neck, anterior trunk, and both upper extremities. The injury occurred on 31 May 2025 at approximately 16:30 while the patient and a colleague were cleaning a computer using compressed air.

At admission, the total burn extent was assessed as 23% TBSA, first- and second-degree deep partial-thickness burns were present (with 1% corresponding to 175 cm^2^), and the patient was initially hospitalised at the standard ward. A subsequent reassessment revised the total burn extent to 31% TBSA, second-degree superficial partial thickness and deep partial-thickness burns. To support the healing of the burned areas and to improve the local wound condition, the patient was indicated for hyperbaric oxygen therapy (HBO). An ENT evaluation performed prior to HBO revealed no contraindications.

The patient underwent regular dressing changes under pre-medication and participated in rehabilitation. Progressive separation of necrotic tissue was observed, and the wounds showed favourable spontaneous healing without the need for surgical intervention. The hospital stay was uneventful, without local or systemic complications.

At discharge, the burned areas on the face, neck, anterior trunk, and both upper extremities were healed, with only minimal residual defects left for short-term outpatient management. The patient was released in a stable and clinically compensated condition.

**Figure 10:**
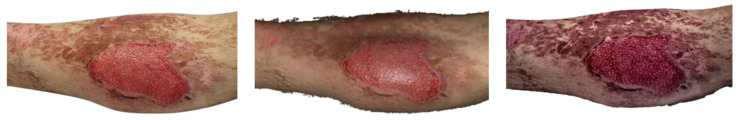
Photograph (**left**), Artec Eva 3D scan (**middle**), and Revopoint Miraco 3D scan (**right**) of the burn on the 10th day after its occurrence.

The medial aspect of the right lower leg (Figure 10), specifically medial calf region, inspected on 10 June, ten days post-injury, shows that a second-degree superficial partial thickness burn is present. In the clinical photograph, the wound shows epidermal loss, with small epithelialisation islands already forming within the wound bed.

The Artec Eva scan depicts the same second-degree superficial partial thickness burns on the medial calf, with epithelialisation islands clearly visible. In the inferior portion of the wound, a pink, re-epithelialised area is evident. The surrounding skin displays brownish patches consistent with superficial burn injury, correlating well with the clinical photograph.

The Revopoint Miraco scan likewise shows a second-degree superficial partial thickness burn of the medial calf, again with visible epithelialisation islands. However, the wound appears darker overall, and the surrounding superficially burned skin is rendered with deeper pigmentation, compared to that in the clinical image.

In the clinical photograph taken on 16 June 2025, a re-epithelialised burn wound is visible, with well-defined margins and clearly identifiable sources of epithelialisation arising from cutaneous adnexal structures.

The Artec Eva scan shows a re-epithelialised wound in the same location; however, the surface texture appears less sharply rendered, giving the wound a more uniform appearance.

**Figure 11:**
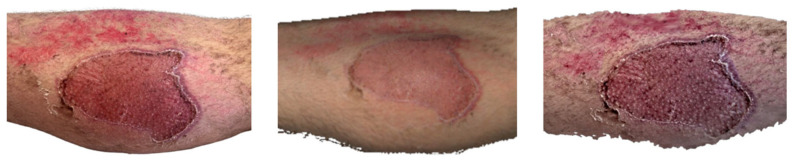
Photograph (**left**), Artec Eva 3D scan (**middle**) and Revopoint Miraco 3D scan (**right**) of the burn on the 16th day after its occurrence.

The Revopoint Miraco scan also demonstrates a re-epithelialised wound, with sources of epithelialisation from cutaneous adnexa clearly visible, similar to the clinical image. The colour representation is darker, creating a slightly different overall impression compared with the photograph and the Artec Eva scan (Figure 11).

**Figure 12:**
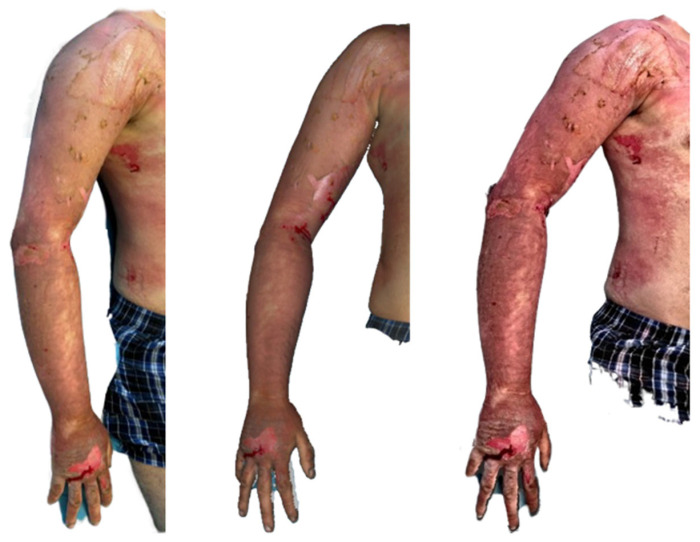
Photograph (**left**), Artec Eva 3D scan (**middle**) and Revopoint Miraco 3D scan (**right**) of the burn on the 4th day after its occurrence.

The upper left arm of the same patient was also inspected (Figure 12). In the clinical photograph taken on 4 June 2025, second-degree superficial partial-thickness burns are present, with epidermal loss visible over the shoulder and the dorsum of the hand. The wound bed is vital, displaying a healthy red appearance.

The Artec Eva scan shows epidermal loss on the shoulder and the dorsum of the hand with a viable wound base, findings that correspond well to the clinical photograph.

The Revopoint Miraco scan likewise demonstrates a second-degree superficial partial-thickness burn on the right upper limb, with the distribution of areas lacking epidermis correlating with the photographic image. The surrounding skin appears darker on this scan, resulting in a slightly different visual impression.

**Figure 13:**
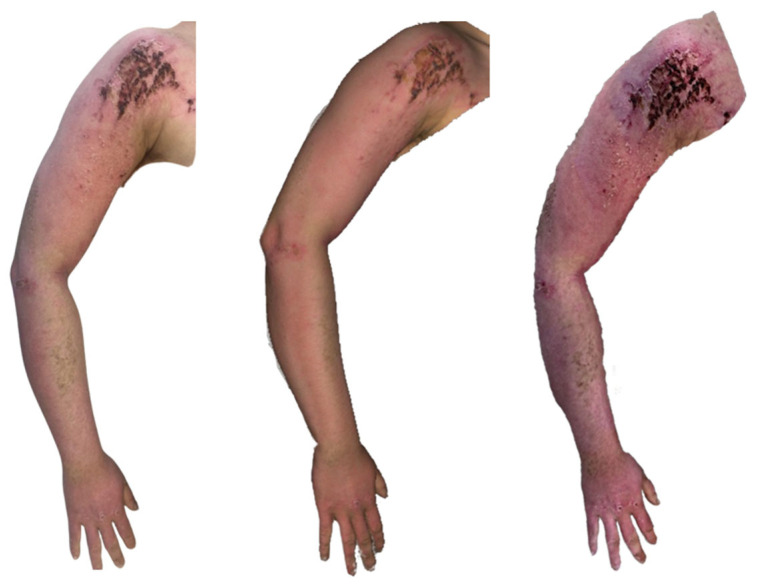
Photograph (**left**), Artec Eva 3D scan (**middle**), and Revopoint Miraco 3D scan (**right**) of the burn on the 10th day after its occurrence.

In the follow-up photographs (Figure 13) obtained six days later (10 June 2025), the previously observed second-degree superficial partial-thickness burn on the right shoulder is shown to have progressed into the crusting phase. The wound surface is now covered by dry, adherent crusts, indicating ongoing maturation of the re-epithelialised tissue beneath. No areas of epidermal loss are newly visible, and there are no signs of infection, maceration, or delayed healing. The surrounding skin appears calm, with only minimal reactive erythema.

On the Artec Eva scan, the affected area is similarly represented with well-demarcated crust formations, consistent with physiological desiccation of the superficial layers during late healing. The surface texture is reproduced in moderate detail, and the overall finding correlates with the clinical image.

The Revopoint Miraco scan also shows the region covered with dry crusts, although the surrounding skin tone appears darker, producing a more contrasting appearance compared with both the photograph and the Artec Eva reconstruction. The distribution and extent of the crusted areas, however, remain consistent across all modalities.

**Figure 14:**
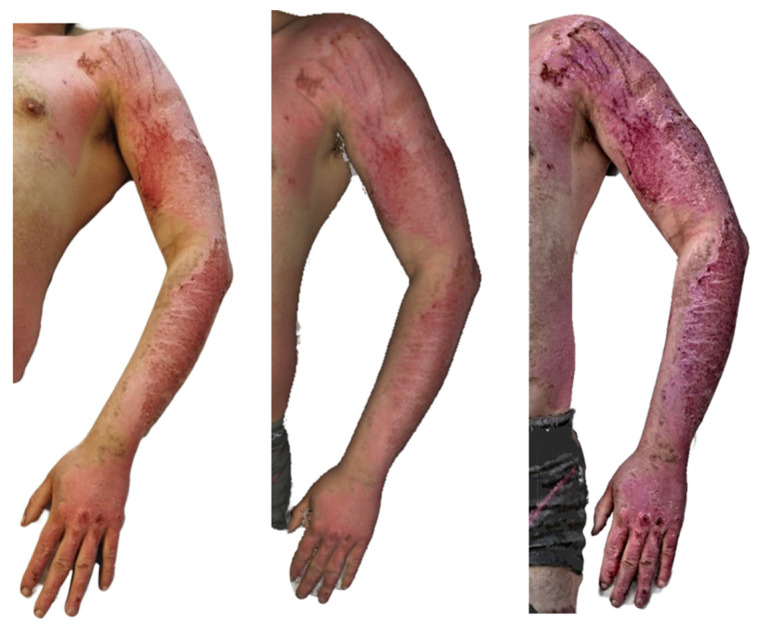
Photograph (**left**), Artec Eva 3D scan (**middle**) and Revopoint Miraco 3D scan (**right**) of the burn on the 10th day after its occurrence.

On 10 June 2025 (day 10 post-injury), in the clinical photograph, the burn on the left upper limb (Figure 14) appears almost completely re-epithelialised. A more erythematous area persists on the shoulder, representing a small residual surface still in the late phase of healing. On the forearm, dry, re-epithelialised skin is present, consistent with the expected maturation stage.

The Artec Eva scan demonstrates a similarly re-epithelialised burn surface, with the shoulder region appearing fully healed. On the forearm, areas of dry skin are visible, though rendered with less surface detail than in the clinical photograph.

The Revopoint Miraco scan likewise shows healed post-burn surfaces on the left upper limb; however, the colour rendering is darker, giving the skin a more intense appearance. A higher amount of dry surface skin is captured, particularly on the forearm. The shoulder area appears more intensely red, with the scan suggesting a greater degree of superficial vascularity, which differs somewhat from the clinical photograph.

## 4. Discussion

The primary objective of this study was to conduct a visual and descriptive comparison of two handheld 3D scanning systems with substantially different technological complexity and cost, and to evaluate their ability to capture clinically relevant features of burn wounds in comparison with standard clinical photography. The focus on visual interpretation reflects routine clinical practice in burn care, where wound appearance, tissue vitality, and dynamic healing changes are primarily assessed visually during bedside examination. From a clinical perspective, the ability to reliably document and visually monitor burn wounds using accessible imaging technologies may support follow-up, interdisciplinary communication, and educational purposes.

Across all documented cases, clinical photographs and 3D scans offered complementary insight into burn wound characteristics, including tissue vitality and healing progression. Second-degree partial-thickness burns showed a consistent pattern of epidermal loss followed by granulation and re-epithelialisation, with 3D reconstructions closely mirroring bedside findings. These observations support the use of 3D surface imaging as a practical adjunct to routine clinical assessment.

When comparing the two scanning systems, Artec Eva provided more reliable colour accuracy, clearer delineation of wound margins, and better visualisation of epithelialisation islands and viable tissue. Subtle surface changes, such as early crusting or fine desquamation, were reproduced more faithfully, which may be clinically relevant for monitoring early healing dynamics. Revopoint Miraco generally correlated with clinical photography in identifying the extent of injury but frequently displayed darker colour tones, enhancing contrast and occasionally giving the false impression of deeper or more heterogeneous tissue involvement. During later healing stages, it often exaggerated surface dryness or surface relief, requiring more cautious visual interpretation. Despite these differences, both systems consistently captured key healing features, including areas of epidermal loss, necrotic surfaces in deeper burns, epithelialisation fronts originating from adnexal structures, crust formation, and fully re-epithelialised areas. Overall, 3D scanning proved to be a valuable adjunct to standard clinical photography. While Artec Eva delivered more visually stable and clinically accurate results, Revopoint Miraco was useful for morphological assessment but required awareness of its colour-rendering limitations.

The findings of the present study are consistent with those of previously published work investigating the role of 3D imaging in burn care. Bednarčíková et al. and Ondrejová and Michalíková [4] demonstrated that 3D scanning enhances visualization of wound morphology and supports longitudinal monitoring of burn progression. Similarly, our results confirm that three-dimensional surface models provide complementary visual information rather than replacing conventional clinical assessment. In contrast to studies focusing on objective burn degree determination using advanced non-invasive imaging modalities, such as hyperspectral imaging described by Li et al. [10], the present study did not aim to determine burn severity based on physiological or perfusion-related parameters. Instead, it focused on the practical visual performance of handheld scanners under standard clinical conditions. Other studies, such as those by Chang et al. [5], have explored quantitative applications of three-dimensional imaging, including surface-area measurements using LiDAR technology and deep learning approaches. Quantitative analysis was intentionally excluded from the present study, as the evaluated devices are not primarily intended for validated medical quantification.

This study has several limitations. First, the number of evaluated cases was limited, and only a subset of patients was selected for detailed analysis. Second, the study was qualitative in nature and did not include quantitative comparisons, such as surface area measurements or objective colorimetric analysis. Third, image acquisition and interpretation were dependent on operator performance and experience, which may influence visual outcomes.

Additionally, similar to other studies in this field, standard reference methods such as laser Doppler imaging (LDI) were not used for determining burn degree or for validating visual findings. As a result, conclusions are limited to visual correspondence with clinical photography rather than objective confirmation of burn severity. These limitations reflect common challenges in current clinical applications of three-dimensional imaging and should be addressed in future studies involving larger cohorts, standardized reference modalities, and combined qualitative and quantitative evaluation approaches.

## 5. Conclusions

This series of burn cases demonstrates that 3D surface scanning is a valuable adjunct to standard clinical photography, providing objective visualisation of wound morphology and healing progression. Both scanning systems were able to reliably identify the essential features of burn injuries, including the extent of epidermal loss, presence of necrotic tissue, and gradual re-epithelialisation.

Across all cases, the Artec Eva scanner delivered more consistent and clinically accurate representations of wound colour, texture, and tissue vitality. In contrast, Revopoint Miraco produced generally usable but darker and more contrast-enhanced images, which in some instances required cautious interpretation.

Despite these differences, both devices successfully supported clinical evaluation and documentation. Their complementary use has the potential to improve monitoring of burn healing, enhance objectivity in follow-up assessments, and contribute to more precise comparison of wound status over time

## Data Availability

The original contributions presented in this study are included in the article. Further inquiries can be directed to the corresponding author.
